# Development of a Novel, Ultra-rapid Biosensor for the Qualitative Detection of Hepatitis B Virus-associated Antigens and Anti-HBV, Based on “Membrane-engineered” Fibroblast Cells with Virus-Specific Antibodies and Antigens

**DOI:** 10.3390/s90302176

**Published:** 2009-03-25

**Authors:** Antonios Perdikaris, Nikos Alexandropoulos, Spiridon Kintzios

**Affiliations:** 1 Laboratory of Plant Physiology, Faculty of Biotechnology, Agricultural University of Athens, Iera Odos 75, 11855 Athens, Greece; E-Mail: antonis@biotechmail.com (A.P.); 2 Hippokration General Hospital, Microbiology Division, Vas. Sofias Av. 114, Athens, Greece; 3 EMBIO Diagnostics Project, Nicosia, Cyprus; E-Mail: info@embio.org (N.A.)

**Keywords:** Bioelectric Recognition Assay, Cell biosensor, Membrane-engineering, Hepatitis viruses, Vero cells

## Abstract

A novel miniature cell biosensor detection system for the detection of Hepatis B virus (HBV)-associated antigens and anti-HBV is described. The biosensor is based on “membrane-engineered” Vero fibroblast cells immobilized in an alginate matrix. The membrane-engineering process involved the electroinsertion of anti-HBV specific antibodies (anti-HBs, anti-HBe) or antigens (HBsAg) in the membranes of the Vero cells. The attachment of a homologous antigen to the electroinserted antibody (or, respectively, of the antibody to the electroinserted antigen) triggered specific changes to the cell membrane potential that were measured by appropriate microelectrodes, according to the principle of the Bioelectric Recognition Assay (BERA). The sensor was used for screening 133 clinical blood serum samples according to a double-blind protocol. Considerably higher sensor responses were observed against HBV-positive samples, compared with responses against negative samples or samples positive for heterologous hepatitis viruses such as Hepatitis C (HCV) virus. Detection of anti-HBs antibodies was made possible by using a biosensor based on immobilized Vero cells bearing the respective antigen (HBsAg). The observed response was rapid (45 sec) and quite reproducible. Fluorescence microscopy observations showed that attachment of HBV particles to cells membrane-engineered with anti-HBs was associated with a decrease of [Ca^2+^]cyt. The perspectives for using the novel biosensor as a qualitative, rapid screening, high throughput assay for HBV antigens and anti-HBs in clinical samples is discussed.

## 1. Introduction

Hepatitis B virus is one of the most widespread viruses, especially (though not only) in developing countries and is an important cause of acute and chronic infection of the liver. The incubation period of hepatitis B varies from 1 to 6 months. Basic markers of virus replication in serum include the S1 proteins of the hepatitis B surface antigen (HBsAg) and a soluble antigen, hepatitis B e antigen (HBeAg), which is secreted by infected hepatocytes [1].

HBsAg appears during infection and is used mainly to screen for the presence of the infection. During the first stages of infection HBsAg is not always present or is not detectable later in the infection, as in many cases it is being cleared by the host, that is, if the host is able to clear the infection, HBsAg will not be detectable. At this phase antibodies anti-HBs and anti-HBc will still be present.

Due to the complexity of the infection process the evaluation of the test results is not always an easy procedure. For example, a person negative for HBsAg and positive for anti-HBs may have cleared an infection or a vaccination has taken place [2]. The fast detection of hepatitis viruses is still a challenging issue for the developers of diagnostic systems, facing challenges associated with increasing demands, difficulties in recruiting an appropriately skilled work force and pressure to improve turnaround times. In particular, automated immunochemical systems are characterized by high throughput and selectivity, but often compromising sensitivity and speed, with the time required to get results ranging from 2 to 26 days [3]. Nucleic acid technologies are quite specific and sensitive but require time and investment in laboratory infrastructure and staff training [4]. In recent years there has been a rapid increase in the number of diagnostic applications for HBV based on biosensors. Various technologies have been used for this purpose. Electrochemical sensors voltametrically detect DNA sequences related to HBV [5–8], while sequence-specific detection has also been achieved by piezoelectric sensors [9–12] and impedance spectroscopy [13]. Detection of HBV antibodies in serum has been facilitated by surface plasmon resonance (SPR) [14] and chemiluminescent immunoassays [15]. Representing the most advanced (and complicated) biosensor technology, cell-based sensors utilize the measurement of whole cell responses to target compounds, such as oxygen consumption, surface chemical or electrical potential, mobility or genetic activity [16].

In the present report, novel cell sensors were developed for the detection of HBV and anti-HBs in clinical samples. The sensors were based on the Bioelectric Recognition Assay (BERA), originally developed by Kintzios *et al*. [17] as a novel method for the detection of plant and human viruses. The assay is based on a specific interaction between the examined virus protein and the immobilized mammalian cells of the sensor causing the change of the electric potential across the cell membrane. Moschopoulou *et al*. [18] recently developed a new methodological approach for increasing the selective response of cellular sensors against specific viral proteins. This approach is based on a membrane-engineering process involving the electroinsertion of virus-specific antibodies in the membranes of fibroblast cells. As a representative example, Vero fibroblasts were engineered with antibodies against *Cucumber mosaic virus* (CMV). The attachment of a homologous virus triggered specific changes to the cell membrane potential, while no change was observed upon cell contact with the heterologous *Cucumber Green Mottle Mosaic Virus (CGMMV).* The same approach was used in the present study, where anti-HBV specific antibodies (anti-HBs, anti-HBe) or antigens (HBsAg) were electroinserted in the membranes of the Vero cells in order to establish a selective response against HBV or anti-HBs, respectively.

## 2. Experimental Section

### 2.1. Materials

Vero cell cultures were originally provided from LGC Promochem (Teddington, UK). Bovine serum albumin (BSA), l-glutamine, propidium iodide and Fluo-3 were purchased from Invitrogen (Carlsbad, CA, USA). All other reagents were purchased from Fluka (Buchs, Switzerland). HBV mouse polyclonal antibodies (anti-HBs, anti-HBe) and antigens (HBsAg) were provided by Abbott Diagnostics Division (Illinois, USA) and prepared in solutions of 0.0125 μg/mL, 0.01 μg/mL and 0.0125 μg/mL, respectively, diluted in saline buffer 0.5% (w/v). Cells were cultured according to procedure previously described by Moschopoulou *et al*. [18]

### 2.2. Sensor Fabrication from Vero Cells

Three series of membrane-engineered cells were created by electroinserting HBV mouse polyclonal antibodies (anti-HBs (A), anti-HBe (B) sensor series) and antigen (HBsAg (C) sensor series), into the membrane of Vero cells following a modified protocol as described by Moschopoulou *et al*. [18]. Specifically the electroinsertion was performed by applying two square electric pulses at 400 V/cm. Following the procedure, approximately 12 × 10^3^ antibodies or antigens, respectively, were incorporated on the surface of each membrane engineered cell, as estimated by immunological assays (data not shown). Engineered Vero cells were mixed with 3 mL of 4% (w/v) sodium alginate solution and then the mixture was added dropwise, by means of a 22G syringe, to 0.8 M CaCl_2_. Each of the resulting calcium alginate beads had an approximate diameter of 2 mm and contained approximately 4 × 10^4^ cells (as determined by optical microscopy). Following this procedure, a batch of 100 consumable sensors was prepared in three hours.

### 2.3. Sample Preparation

Blood serum samples were provided from Hippokration General Hospital in Athens, Greece and stored at −5 °C. All samples have been tested for HBsAg, HBeAg, anti-HBsAg, anti-HCV and anti-HAV IgM using the Abbot AxSYM^®^ test. The samples were also assayed for HCV using the Amplicor HCV Monitor v2.0 (Roche). No quantitative information (virus titer, viral load) was provided. This was in agreement with our primary goal to develop a qualitative only cell sensor for HBV. Prior to the assay, samples were defrosted and kept in room temperature at 20 °C. An additional 35 blood serum samples were used as negative samples, after being tested for hepatitis viruses’ presence or respective antibodies (Table 1).

### 2.4. Assay Procedure

**Figure 1.**
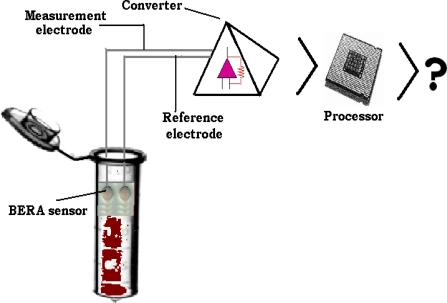
Schematic representation of the immobilized cell biosensor. The reference electrode is manually inserted in a cell-free gel bead, while the measuring electrode is inserted in the immobilized cell-loaded gel bead. Neither electrode is in direct contact with the sample solution (i.e. only beads are immersed into sample). The approximate diameter of the beads is two mm. Both measurement and reference electrodes are connected to the PMD 1608-FS converter.

Following the procedure as described by Mavrikou *et al*. [19], each cell sensor was connected to a working electrode made from pure silver, electrochemically coated with an Ag/AgCl layer and having a diameter of 0.75 mm. Electrodes were connected to a PMD-1608FS A/D card (Measurement Computing, Middleboro, MA). Signal and data processing were recorded with InstaCal software (Measurement Computing). For each assay, the sensor system (presented in Figure 1) was immersed into each sample solution (400 μL). The response of each sensor was estimated by recording the maximum value of the sensor potential for a period of 45 sec after sample application.

### 2.4. Fluorescence Microscopy

Changes in cytoplasmic Ca^2+^ concentration in cells membrane-engineered with anti-HBs (A), anti-HBe (B) and HBsAg (C) before and after the addition of HBsAg (0.0125 μg/mL), HBeAg (0.01 μg/mL) and anti-HBs (0.0125 μg/mL), respectively, were monitored by the uptake of the acetomethyl ester of Fluo3 [18,20]. After application of five μl of the dye, the fluorescence of the specimens was recorded for five minutes at 10 s intervals. Slides with stained cells were mounted on a Zeiss Axiolab fluorescent microscope equipped with a BP-546 excitation filter and an FT-580 chromatic beam splitter. In order to control photobleaching, we kept specimen exposure times at a minimum level. No significant alteration of the intensity of the fluorescence was observed during the observation of the specimens.

### 2.5. Experimental Design

Experiments were set-up in a completely randomized design. Clinical sample analysis was conducted according to a double-blind protocol. For the detection process 133 different serum samples were used (98 positive, 35 negative), according to the classification presented in Table 1. Each sample was tested with the three different types of sensors (A, B and C). For each test, each sample was assayed five times, i.e. with five individual, different biosensors. The assay experiment was repeated three times, therefore each sample was actually assayed with 15 different sensors. In fluorescent microscopy assays, the number of cells (fluorescent or not) was counted both manually and by using Doc-It® LS Image Acquisition and Analysis Software. For each microscopic observation, the average cell number and fluorescence assays were calculated from 10 different 7 × 10^3^ μm^2^ optical fields.

## 3. Results

### 3.1. Responses of Biosensors Based on Cells Membrane-engineered with Anti-HBs, Anti-HBe or HBsAg

**Figure 2.**
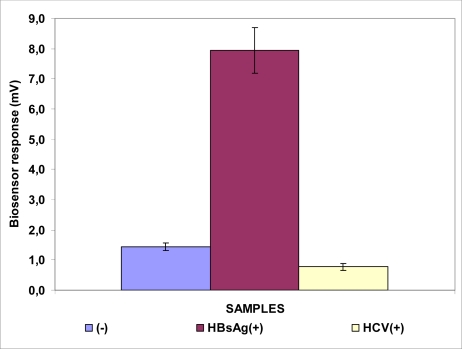
Biosensor response against HBsAg. The sensor is based on Vero cells membrane-engineered with the anti-HBs antibody. Sensor response is expressed as an associated change in the membrane potential of immobilized cells. Error bars represent standard errors of the average value of all replications with each sample (*n* = 15 replications for each sample).

**Figure 3.**
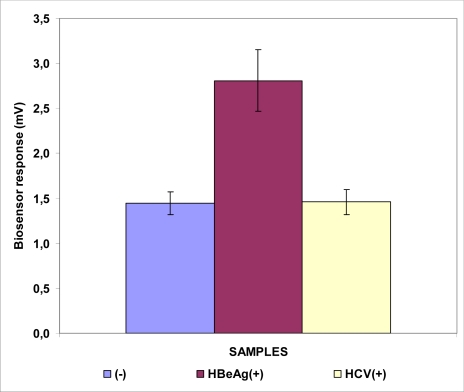
Biosensor response against HBeAg. The sensor is based on Vero cells membrane-engineered with the anti-HBe antibody. Sensor response is expressed as an associated change in the membrane potential of immobilized cells. Error bars represent standard errors of the average value of all replications with each sample (*n* = 15 replications for each sample).

**Figure 4.**
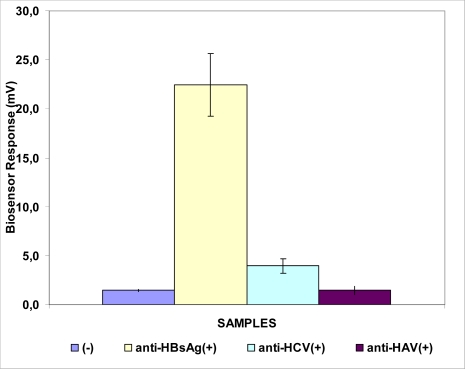
Biosensor response against the anti-HBs antibody. The sensor is based on Vero cells membrane-engineered with HBsAg. Sensor response is expressed as an associated change in the membrane potential of immobilized cells. Error bars represent standard errors of the average value of all replications with each sample (*n* = 15 replications for each sample).

The responses of the BERA cell sensors based on membrane-engineered Vero cells are presented in Figures 2–4. The response of the biosensor based on Vero cells bearing the anti-HBs antibody (Sensor A) to samples positive for the HBsAg (7.1 ± 0.8 mV) was considerably higher compared to samples containing heterologous hepatitis viral antigens (HCV) or negative samples (Figure 2). The response of the sensor was also quite reproducible, with a variation of 8.9%.

A similar result, but with a considerably lower intensity of response (3.5 ± 0.6 mV, Figure 3) to samples positive for the HBeAg was obtained with the biosensor based on Vero cells bearing the anti-HBe antibody (Sensor B). As in the case of HBsAg detection, the response was considerably higher compared to samples containing heterologous hepatitis viral antigens (HCV) or negative samples.

Detection of anti-HBs antibodies in clinical samples was made possible by using a biosensor based on immobilized Vero cells bearing the respective antigen (HBsAg) (Sensor C). A very high response of the sensor to samples positive for anti-HBs (23 ± 4 mV) was observed compared to samples containing antibodies of the heterologous viruses (anti-HAV, anti-HCV) or negative samples (Figure 4).

### 3.2. Fluorescence Microscopy Assays of Cell–virus Interactions

**Figure 5.**
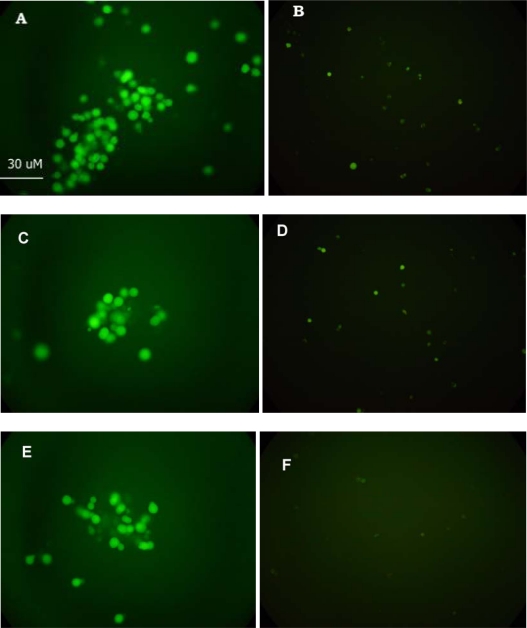
Changes (expressed as differences in fluorescence intensity) of the cytoplasmic calcium ion concentration in Vero cells, membrane-engineered with anti-HBs (A, B), anti-HBe (C, D) and HBsAg (E, F), before (A, C, E) and after (B, D, F) treatment with the HBsAg, HBeAg and anti-HBs respectively.

Vero cells membrane-engineered with anti-HBs responded to the presence of the homologous antigen (HBsAg) by a rapid and very considerable depletion of their intracellular Ca^2+^ stores (Figure 5). Similar responses were obtained after treating the anti-HBe and HBsAg engineered Vero cells with the homologous antigen and antibody, respectively. No changes were observed in fluorescence density, when cells treated with a virus-free solution.

## 4. Discussion

The results of the present study demonstrate that it is possible to detect HBV-associated antigens and antibodies with whole cell sensors based on mammalian cells membrane-engineered with the respective receptor-like molecule. One of the main assumptions associated with the use of membrane-engineered cells as recognition elements in biosensors is that, the interaction of viral particles/antibodies with electroinserted antibodies/antigens will cause a measurable change of the cell membrane potential [18,21]. In our experiments, the considerable cell membrane hyperpolarization and the observed decrease of cytoplasmic Ca^2+^ concentration after treating membrane-engineered Vero cells with the homologous target analytes, but not with control samples or heterologous samples, provides a supporting indication for this hypothesis. Whelan and Zare [20] have previously shown that receptor-like interactions between molecules on the cell surface and target analytes resulted in a detectable change in the concentration of cytosolic Ca^2+^. In this way, cells with electroinserted antibodies serve as recognition platforms for homologous viruses, whereas the translation of the recognition reaction into potential changes makes the assay much more rapid (and, in some cases, more sensitive) than conventional immunoanalytical methods. Of course, the selectivity of membrane-engineered cell sensors strongly depends on the specificity of the embedded antibodies (or antigens, in the case of anti-HBs detection). Therefore, the present methodological approach shares some of the limitations present in enzyme immunoassays. On the other hand, the sensor response is independent from factors narrowing the applicability of plate-trapped antigen (PTA) immunoassays, such as the duration of incubation time [22].

The sensor was successfully used to detect HBV and its antibodies in clinical samples in an selective and satisfactory reproducible (11–17% variation) manner. HBV detection with advanced biosensor systems is rapidly gaining in favour of conventional methods. For instance, recently reported immunosensors are able to detect HBsAg and/or anti-HBs at nano-[13,14] or even picomolar concentrations [12,15] at a considerably faster rate of analysis. Typical assay procedures include sample injection and alternating washing/incubation steps, with a total duration between 30 and 200 min. Our novel sensor is able to perform even faster (total assay time: 45 sec) with an equally satisfactory clinical specificity. Compared to optical and electrochemical sensors, the novel system has the additional advantage of a simpler, one-step, label-free assay format with minimum cross-reactivity. As any novel system, the sensor described in the present study constitutes a preliminary step towards the development of an operational HBV detection tool based on membrane-engineered cells. In accordance with other immunosensor technologies, the sensitivity of the system for HBV-associated antigen/antibody detection is 10–100 fold lower than with sensors utilizing nucleic acid sequences [11–13,23]. From a limited number of comparative tests (25 samples-analytical results not shown) between the novel biosensor and real time PCR, we know that the sensitivity of the sensor is at the level of 10^3^ viral copies/ml. In terms of conventional immunological assays (ELISA), the detection limit of HBsAg/HBeAg is approximately 0.05 ng/mL and the detection limit of HBe antigen is less that 1 PEI (Paul Ehrlich Institute reference serum) U/μL. Of course more extensive, comparative studies are required in order to determine the actual detection limit. Further increase in the sensor sensitivity could arise by increasing the concentration of immobilized cells per sensor or the concentration of electroinserted antibodies onto the cell surface. It may also not be possible to use the sensor for quantitative virus detection. As previously reported [18], molecular recognition of virus particles by membrane-engineered cells is based on an electromechanical stress at the cite of virus-antibody reaction on the membrane, leading to changes in membrane porosity and, in consequence, membrane conductivity. This reaction is non-titrimetric, so that lack of linearity in the sensor response against different virus concentrations maybe expected. Although quantitative virus detection is always desired, this limitation is not important in the context of using the novel sensor as a qualitative, rapid screening, high throughput assay. Using a multiple cell-electrode interface array (currently under testing by our research group), it is possible to conduct more than 1,150 individual tests/h, a capacity considerably larger than with conventional immunoassay systems (80–100 test/h) [24]. In addition, the novel cell biosensor has the advantage of increased storability, since immobilized, membrane-engineered cells can be maintained alive for up to four weeks after provision of 20 (v/v) fetal calf serum [25].

## Figures and Tables

**Table 1.** Classification of serum samples used.

**Sample type**	**Number of samples**
HBsAg(+), anti-HBsAg(+)	35
HBeAg(+)	25
HCV(+), anti-HCV(+)	32
anti-HAV IgM(+)	6
Total positive	98
Negative	35
Total	133
